# Structure-Based Discovery of Small-Molecule Inhibitors of the Autocatalytic Proliferation of α-Synuclein Aggregates

**DOI:** 10.1021/acs.molpharmaceut.2c00548

**Published:** 2022-11-14

**Authors:** Sean Chia, Z. Faidon Brotzakis, Robert I. Horne, Andrea Possenti, Benedetta Mannini, Rodrigo Cataldi, Magdalena Nowinska, Roxine Staats, Sara Linse, Tuomas P. J. Knowles, Johnny Habchi, Michele Vendruscolo

**Affiliations:** † Centre for Misfolding Diseases, Yusuf Hamied Department of Chemistry, University of Cambridge, CambridgeCB2 1EW, U.K.; ‡ Department of Biochemistry & Structural Biology, Center for Molecular Protein Science, Lund University, 221 00Lund, Sweden; § Department of Physics, Cavendish Laboratory, CambridgeCB3 0HE, U.K.

**Keywords:** Parkinson’s disease, α-synuclein, protein aggregation, computational docking, structure-based small-molecule discovery, kinetic-based small-molecule discovery

## Abstract

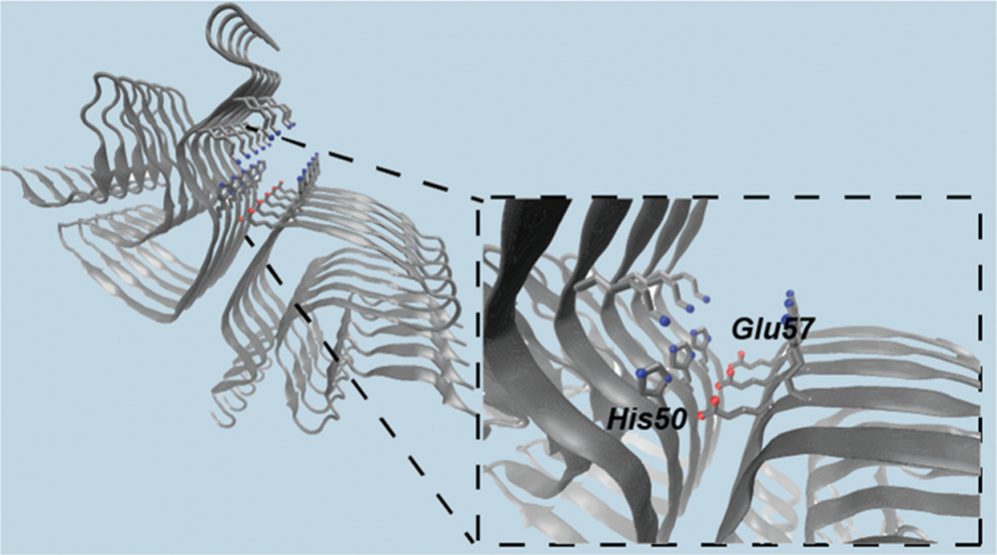

The presence of amyloid fibrils of α-synuclein is closely associated with Parkinson’s disease and related synucleinopathies. It is still very challenging, however, to systematically discover small molecules that prevent the formation of these aberrant aggregates. Here, we describe a structure-based approach to identify small molecules that specifically inhibit the surface-catalyzed secondary nucleation step in the aggregation of α-synuclein by binding to the surface of the amyloid fibrils. The resulting small molecules are screened using a range of kinetic and thermodynamic assays for their ability to bind α-synuclein fibrils and prevent the further generation of α-synuclein oligomers. This study demonstrates that the combination of structure-based and kinetic-based drug discovery methods can lead to the identification of small molecules that selectively inhibit the autocatalytic proliferation of α-synuclein aggregates.

## Introduction

Parkinson’s disease (PD) is the most common neurogenerative movement disorder, which affects over 6 million individuals worldwide.^1−4^ This disease is characterized histopathologically by the accumulation of aberrant deposits known as Lewy bodies, which are composed primarily of the aggregated form of the intrinsically disordered protein α-synuclein.^5,6^ Aggregates of α-synuclein, including misfolded oligomers and highly ordered amyloid fibrils, can induce neurotoxicity through a multitude of mechanisms, including cell membrane disruption and mitochondrial damage, which ultimately cause neuronal death.^7−9^ In particular, recent evidence implicates prefibrillar α-synuclein oligomeric species in the PD pathology: α-synuclein oligomers appear to specifically interact with the ATP synthase to induce mitochondrial dysfunction, cause early axonal dysfunction, and increase the TLR4-dependent sensitized inflammatory response leading to greater reactive oxygen species (ROS) production.^10−12^

Because of the relevance of α-synuclein aggregates, much effort has been devoted toward the characterization of their structures.^13−17^ These structures have enabled the development of structure-based drug discovery approaches, including in particular the identification of peptide-based inhibitors to prevent α-synuclein aggregation.^18,19^ Furthermore, binding sites along the surface of α-synuclein fibrils for the development of diagnostics tools have also been identified.^20−23^ These developments are relevant considering the current lack of radiotracers for measuring the accumulation of α-synuclein aggregates in the human brain and that such diagnostics tools may eventually enable the presymptomatic diagnosis of synucleinopathies.^20,24−26^

Previous studies have used high-throughput docking approaches to identify α-synuclein fibril-binding compounds.^20−23^ However, because of the great technical difficulties in establishing reproducible high-throughput kinetic assays to monitor α-synuclein aggregation, the experimental validation of the compounds predicted from computational screens has been challenging. Recent advances in chemical kinetics approaches have allowed the identification of small molecules and molecular chaperones that are able to inhibit α-synuclein aggregation.^27−30^ It has thus been possible to inhibit specifically the surface-catalyzed secondary nucleation step, which is responsible for the autocatalytic proliferation of α-synuclein fibrils, by binding competitively with α-synuclein monomers along specific sites on the surface of α-synuclein fibrils.^27,29^ Considering that oligomers are generated primarily by surface-catalyzed secondary nucleation,^31,32^ the discovery of compounds targeting this mechanism offers promising opportunities for drug discovery.^28,33^ This approach is particularly advantageous as it allows experiments to be performed in a high-throughput manner in 96-well plates, while conferring quantitative analysis of the effect of the compounds on specific microscopic steps in the aggregation process of α-synuclein. These quantitative measurements subsequently enable structure–activity relationship (SAR) studies and facilitate the systematic optimization of the compounds’ properties.^28,33^

In the present study, we identified compounds that bind to α-synuclein fibrils to make advances on two problems: (1) how to prevent the fibril-catalyzed secondary nucleation in the autocatalytic proliferation of α-synuclein fibrils, and (2) how to identify molecular tracers for measuring the accumulation of α-synuclein aggregates through imaging methods. In an initial step toward these goals, we demonstrate the use of an *in silico* and *in vitro* combinatorial framework in identifying compounds that bind to α-synuclein fibrils and subsequently inhibit the secondary nucleation process in the aggregation of α-synuclein. In this framework, we first employ a computational method that combines two docking techniques to identify compounds with high predicted binding affinity for α-synuclein fibrils. This list is then validated experimentally through chemical kinetics, which identifies the top compounds that are able to inhibit the surface-catalyzed secondary nucleation step in the aggregation of α-synuclein. The binding affinity of these compounds for α-synuclein fibrils is then validated experimentally.

Overall, this strategy demonstrates the rational development of a combined structure-based and kinetic-based framework to identify compounds that can bind to α-synuclein fibrils and presents an opportunity for the systematic development of compounds that can be potentially used as therapeutic and diagnostic tools for synucleinopathies.

## Materials and Methods

### Computational Docking

The docking protocol used in this study comprises four stages. First, we determined a binding site on the α-synuclein fibrils. To achieve this goal, we analyzed a structure of α-synuclein fibrils (PDB ID: 6cu7) using Fpocket,^34^ which identifies potential binding pockets based on volume criteria (Figure 3F). We found a pocket in the fibril core (surrounded by residues His50–Lys58 and Thr72–Val77) and a pocket on the fibril surface (surrounded by His50 and Glu57). We focused on the surface binding pocket because the buried binding pocket is unlikely to act as a catalytic site for α-synuclein secondary nucleation. Moreover, α-synuclein secondary nucleation has been reported as significant only below pH 5.8,^35^ when histidine residues are protonated, also supporting the choice of the surface binding pocket.

For the selection of screening compounds, we used the ZINC library, which contains a set of over 230 million purchasable compounds for screening.^36^ To prioritize the chemical space of small molecules considered in the docking calculations, central nervous system multiparameter optimization (CNS MPO) criteria^37^ were applied, effectively reducing the space to ∼2 million compounds. In particular, CNS MPO has been shown to correlate with key *in vitro* attributes of drug discovery, and thus using this filter potentially enables the identification of compounds with better physicochemical and pharmacokinetic properties pertaining to brain penetration, where α-synuclein is localized.^37^ We further subjected these compounds to docking calculation against the binding site identified above using AutoDock Vina.^38^ To increase the confidence of the calculations, the top-scoring 10 000 small molecules were selected and docked against the same α-synuclein binding site, using FRED (OpenEye Scientific Software).^39^ The top-scoring, common 1000 compounds in both docking protocols are selected and clustered using Tanimoto clustering,^40^ leading to a list of 79 clusters.

### Preparation of Compounds and Chemicals

The centroids from the above 79 clusters were selected for experimental validation. Compounds were purchased from MolPort (Riga, Latvia), and in the cases for which centroids were not available for purchase, the compounds in the clusters with the closest chemical structures were used as the representative compounds instead. In the end, a total of 67 compounds were purchased (centroids and alternative compounds in 12 clusters were all not available for purchase) and then prepared in DMSO to a stock of 5 mM. All chemicals used were purchased at the highest purity available (>90% in purity).

### Preparation of α-Synuclein

Recombinant α-synuclein was purified as described previously.^35,41,42^ The plasmid pT7-7 encoding for human α-synuclein was transformed into chemically competent *E. coli* cells of strain BL21 (DE3)-gold cells (Thermo Scientific). Following transformation, the cells were grown in lysogeny broth (LB) in the presence of ampicillin (100 μg/mL). Cells were induced with isopropyl β-d-1-thiogalactopyranoside (IPTG), grown overnight at 37 °C for 4 h, and harvested by centrifugation in a Beckman Avanti J25 centrifuge with a JA-20 rotor at 5000 rpm (Beckman Coulter, Fullerton, CA). The cell pellet was resuspended in 10 mM Tris, pH 8.0, 1 mM EDTA, 1 mM PMSF and lysed by multiple freeze–thaw cycles and sonication. The cell suspension was boiled for 20 min, and then cooled and centrifuged at 13 500 rpm with a JA-20 rotor (Beckman Coulter). Streptomycin sulfate was added to the supernatant to a final concentration of 10 mg/mL and the mixture was stirred for 15 min at 4 °C. After centrifugation at 13 500 rpm, the supernatant was supplemented with 0.36 g/mL ammonium sulfate. The solution was stirred for 30 min at 4 °C and centrifuged again at 13 500 rpm. The pellet was resuspended in 25 mM Tris, pH 7.7, and ion-exchange chromatography was performed using an HQ/M-column in buffer A (25 mM Tris, pH 7.7) with a linear gradient to buffer B (25 mM Tris, pH 7.7, 600 mM NaCl). The fractions containing α-synuclein (≈300 μM) were dialyzed overnight against the appropriate buffer. The protein concentration was determined from the absorbance at 280 nm using the extinction coefficient ε_280_ = 5600 M^–1^ cm^–1^.

### Preparation of α-Synuclein Fibril Seeds

α-synuclein fibril seeds were produced as described previously.^35,41^ Samples of α-synuclein (700 μM) were incubated in 20 mM phosphate buffer (pH 6.5) for 72 h at 40 °C and stirred at 1500 rpm with a Teflon bar on an RCT Basic Heat Plate (IKA, Staufen, Germany). Fibrils were then diluted to 200 μM, aliquoted and flash frozen in liquid nitrogen, and finally stored at −80 °C. For the use of kinetic experiments, the 200 μM fibril stock was thawed and sonicated for 15 s using a tip sonicator (Bandelin, Sonopuls HD 2070, Berlin, Germany), using 10% maximum power and a 50% cycle.

### Kinetic Assays

α-synuclein was injected into a Superdex 75 10/300 GL column (GE Healthcare) at a flow rate of 0.5 mL/min and eluted in 20 mM sodium phosphate buffer (pH 4.8) supplemented with 1 mM EDTA. The obtained monomer was diluted in buffer to a desired concentration and supplemented with 50 μM ThT and preformed α-synuclein fibril seeds. The compounds (or DMSO alone) were then added at the desired concentration to a final DMSO concentration of 1% (v/v). Samples were prepared in low-binding Eppendorf tubes and then pipetted into a 96-well half-area, black/clear flat-bottom polystyrene NBS microplate (Corning 3881), 150 μL/well, with three replicates per sample ran in parallel (two replicates in the case of the high-throughput screening in Figure 2). The assay was then initiated by placing the microplate at 37 °C under quiescent conditions in a plate reader (FLUOstar Omega, BMG Labtech, Aylesbury, U.K.). The ThT fluorescence was measured through the bottom of the plate with a 440 nm excitation filter and a 480 nm emission filter.

### Transmission Electron Microscopy (TEM)

α-Synuclein samples (10 μM) were prepared and aggregated as described in the kinetic assay, in the absence or presence of 25 μM compound C, without the addition of ThT. Samples were collected from the microplate at the end of the reaction (150 h) into low-binding Eppendorf tubes. They were then prepared on 400-mesh, 3 mm copper grid carbon support film (EM Resolutions Ltd.) and stained with 2% uranyl acetate (wt/vol). The samples were imaged on an FEI Tecnai G_2_ transmission electron microscope (Cambridge Advanced Imaging Centre). Images were analyzed using the SIS Megaview II Image Capture system (Olympus).

### Determination of the Elongation Rate Constant

In the presence of high concentrations of seeds (≈μM), the aggregation of α-synuclein is dominated by the elongation of the added seeds. Under these where other microscopic processes are negligible, the aggregation kinetics for α-synuclein can be described by^35,41^
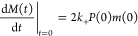
where *M*(*t*) is the fibril mass concentration at time *t*, *P*(0) is the initial number of fibrils, *m*(0) is the initial monomer concentration, and *k*_+_ is the rate of fibril elongation. In this case, by fitting a line to the early time points of the aggregation reaction as observed by ThT kinetics, 2*k*_+_*P*(0)*m*(0) can be calculated for α-synuclein in the absence and presence of the compounds. Subsequently, the elongation rate in the presence of compounds can be expressed as a normalized reduction compared to the elongation rate in the absence of compounds (1% DMSO).

### Determination of the Fibril Amplification Rate Constant

In the presence of low concentrations of seeds, the fibril mass fraction *M*(*t*) over time was described using a generalized logistic function to the normalized aggregation data^28,43^
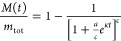
where *m*_tot_ denotes the total concentration of α-synuclein monomers. The parameters *a* and *c* are defined as
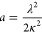

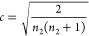
The parameters λ and κ represent combinations for the effective rate constants for primary and secondary nucleation, respectively, and are defined as^28,43^
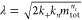

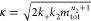
where *k_n_* and *k*_2_ denote the rate constants for primary and secondary nucleation, respectively, and *n_c_* and *n*_2_ denote the reaction orders of primary and secondary nucleation, respectively. In this case, *c* was fixed at 0.3 for the fitting of all data, and *k*_2_, the amplification rate, is expressed as a normalized reduction for α-synuclein in the presence of the compounds compared to in its absence (1% DMSO). The amplification rate *k*_2_ of α-synuclein denotes the generation of fibrillar aggregates through surface-catalyzed secondary nucleation, where monomers nucleate on the surfaces of α-synuclein fibrils.

### Determination of the Oligomer Flux

The prediction of the reactive flux toward oligomers over time was calculated as
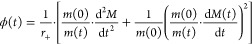
where *r*_+_*=* 2*k*_+_*m*(0) is the apparent elongation rate constant extracted as described earlier and *m*(0) refers to the total concentration of monomers at the start of the reaction.

### Fluorescence Polarization

Compound C (10 μM) was incubated with increasing concentrations of either preformed α-synuclein or Aβ42 fibrils (in 1% DMSO). After incubation, the samples were pipetted into a 96-well half-area, black/clear flat-bottom polystyrene nonbinding surface (NBS) microplate (Corning 3881). The fluorescence polarization of compound C was monitored using a plate reader (CLARIOstar, BMG Labtech, Aylesbury, U.K.) under quiescent conditions at room temperature, using a 360 nm excitation filter and a 430 emission filter. The equilibrium dissociation constant (*K*_d_) was determined by fitting the data to the equation *Y* = *B*_max_ × *X*/(*K*_d_ + *X*) + NS × *X* + background using GraphPad Prism software, where *B*_max_ denotes the maximum specific binding, NS denotes the slope of nonspecific binding per *X* unit, and background denotes the amount of nonspecific binding when no ligand is added.

### Mass Spectrometry

Preformed α-synuclein fibrils (10 μM) were incubated with 10 μM compound C in 20 mM sodium phosphate buffer (pH 4.8) supplemented with 1 mM EDTA overnight under quiescent conditions at room temperature. The samples were then ultracentrifuged at 100 000*g* for 30 min, and the supernatant was removed for analysis using a Waters Xevo G2-S QTOF spectrometer (Waters Corporation, MA).

### Cell Cultures

Human SH-SY5Y neuroblastoma cells (A.T.C.C., Manassas, VA) were cultured in Dulbecco’s modified Eagle’s medium (DMEM)-F12+GlutaMax supplement (Thermo Fisher Scientific, Waltham, MA) with 10% heat-inactivated fetal bovine serum. The cell cultures were maintained in a 5.0% CO_2_ humidified atmosphere at 37 °C and grown until 80% confluence for a maximum of 20 passages.

### Colocalization Assay

Samples containing 100 nM (monomer equivalents) α-synuclein fibrils in 20 mM sodium phosphate buffer, pH 4.8, were pre-incubated in the absence or presence of 1 μM compound C in a 96-well plate. The samples were subsequently stained with pFTAA (Amytracker 630 from Ebba Biotech AB, Sweden). Images were acquired using the fluorescence microscope Cytation5 Cell Imaging Reader (BioTek Instruments, Winooski, VT). For the colocalization assay in the presence of cells, the cells were first plated into a 96-well plate and treated for 24 h with the samples containing 100 nM (monomer equivalents) α-synuclein fibrils in 20 mM sodium phosphate buffer, pH 4.8, in the absence or presence of 1 μM compound C. After incubation, the samples were treated in the same procedure as described above.

## Results

### Framework to Identify Compounds That Bind α-Synuclein Fibrils

**Figure 1:**
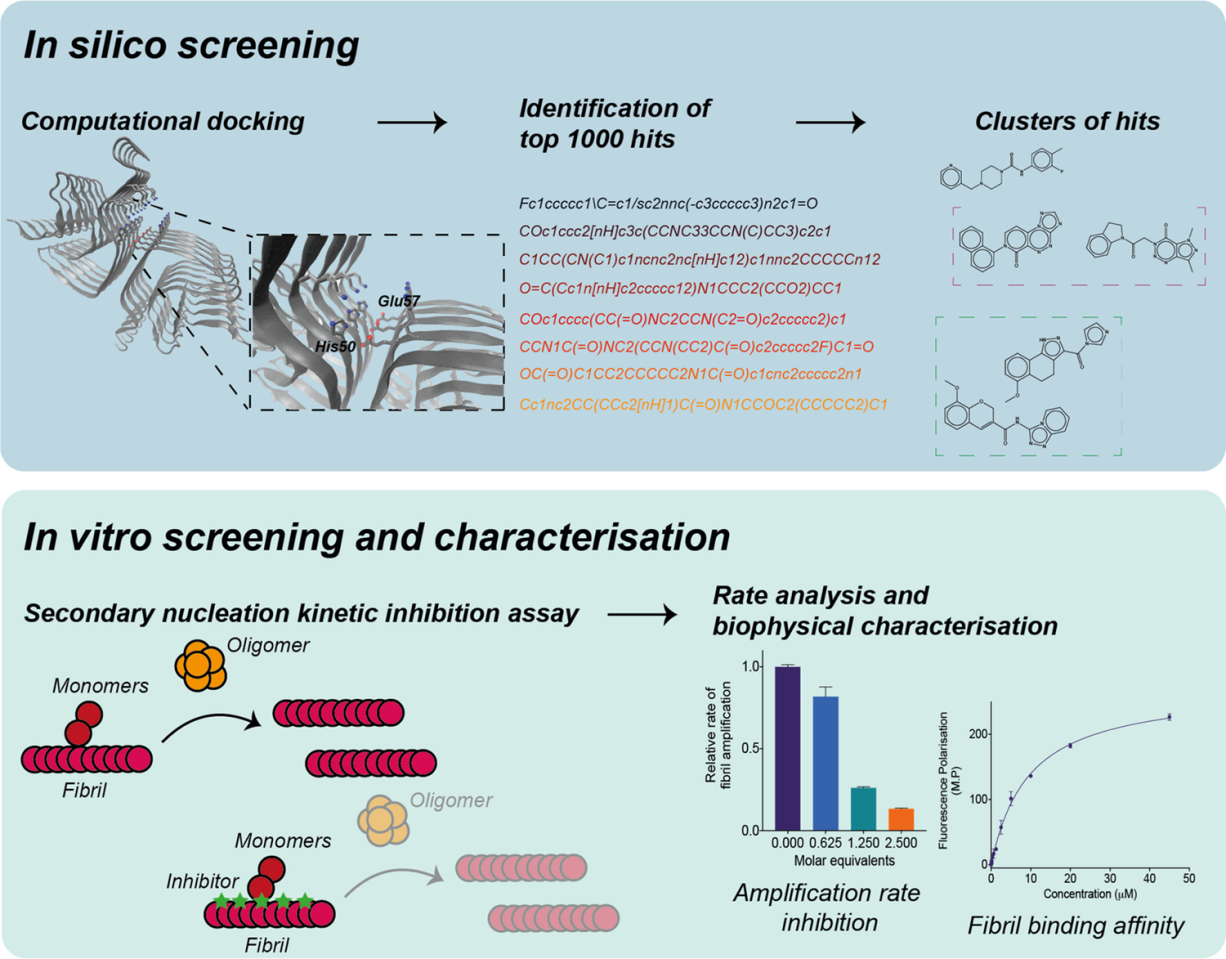
Combined structure-based and kinetic-based approach to identify small molecules that bind α-synuclein fibrils and inhibit its aggregation. In the first step, computational docking is performed on a large library of small molecules. The top candidates are then clustered to identify a subset of chemically diverse compounds that exhibit high predicted binding scores for α-synuclein fibrils. Subsequently, these compounds are experimentally validated through a kinetic assay for their ability to inhibit the secondary nucleation aggregation of α-synuclein by binding to the surface of fibrils. Further rate constant analysis and fibril-binding experiments allow for the positive compounds to be characterized based on both their inhibition of the kinetic assay, as well as their binding affinity toward α-synuclein fibrils.

In this work, we describe a framework to identify compounds that bind specific sites on the surface of α-synuclein fibrils and are able to block the process of fibril-catalyzed secondary nucleation. This method consists of a computational docking approach to identify small-molecule candidates from a large library of compounds, and a subsequent *in vitro* approach based on chemical kinetics to assess the ability of the candidates to inhibit the aggregation of α-synuclein, as well as their affinity toward α-synuclein fibrils (Figure 1).

First, from a library of compounds, small molecules are individually docked against a binding pocket chosen along the groove of the fibril (Materials and Methods section). This groove, which involves residues His50 and Glu57, was selected as the potential site for docking since its geometry, position and physicochemical properties identified it as a likely catalytic site for α-synuclein secondary nucleation (Figure 1 and Materials and Methods section). Using the predicted binding scores (Δ*G*_b_) as the parameter for ranking the compounds, the docking procedure generated a list of top 1000 candidates. As a means of increasing the chemical diversity of the compounds to be experimentally validated, a clustering method based on the chemical similarity of the compounds was adopted, in which the centroids of each of the clusters were selected as the final candidates of the library of compounds to be tested *in vitro* (Figure 1).

Next, these centroid compounds were validated experimentally using a chemical kinetics-based assay of α-synuclein aggregation. Specifically, in the presence of low amounts of preformed seeds and at mildly acidic pH, the aggregation of α-synuclein is dominated by a surface-catalyzed secondary nucleation process, in which monomers form nuclei along the fibril surfaces.^31^ This autocatalytic process results in the rapid generation of aggregates that then elongate to form α-synuclein fibrils. When the aggregation of α-synuclein is performed in the presence of compounds with suitable binding affinity for the surface of the α-synuclein fibrils, this results in a decreased amplification rate of α-synuclein aggregates.^27,29^

The small-molecule inhibitors identified in this way were further validated for their binding affinity toward α-synuclein fibrils using fluorescence polarization and mass-spectrometry-based pull-down assays. Overall, using this framework, small-molecule candidates could be characterized through their predicted and experimental binding affinity, as well as their kinetic inhibitory properties as a result of their interaction with α-synuclein fibrils.

### *In Silico* Docking of Compounds Predicted to Bind α-Synuclein Fibrils

Using AutoDock Vina^38^ and FRED,^39^ a wide distribution of binding scores was observed for compounds in the ZINC library (Materials and Methods).^36^ When assessing the top 10 000 compounds with the highest binding affinity, we observed a wider distribution of binding scores using FRED (−4 to −14 kcal/mol) than AutoDock Vina (−6.6 to −8.6 kcal/mol) (Figure S1). We also found that the predicted binding scores did not correlate strongly between the two methods (Figure S1). Such differences in the predicted scores can be attributed to the different scoring functions used among docking methods, leading to the choice of consensus compounds.^44^ Thus, we selected the top 10% (1000 common compounds) of the candidates with a high predicted binding score in both methods (Figure S1). The candidate library was further refined by employing a clustering method based on the chemical structures of the compounds to identify the centroids of each cluster as the representative compound in the cluster. Although this procedure may result in a lower number of hits due to the exclusion of chemical derivatives of a potential α-synuclein fibril binder that are clustered together, it also increases the chemical diversity of the library set, and therefore allows the sampling of a wider chemical space to screen for potential fibril binders.

### Identification of Compounds That Inhibit α-Synuclein Secondary Nucleation

**Figure 2:**
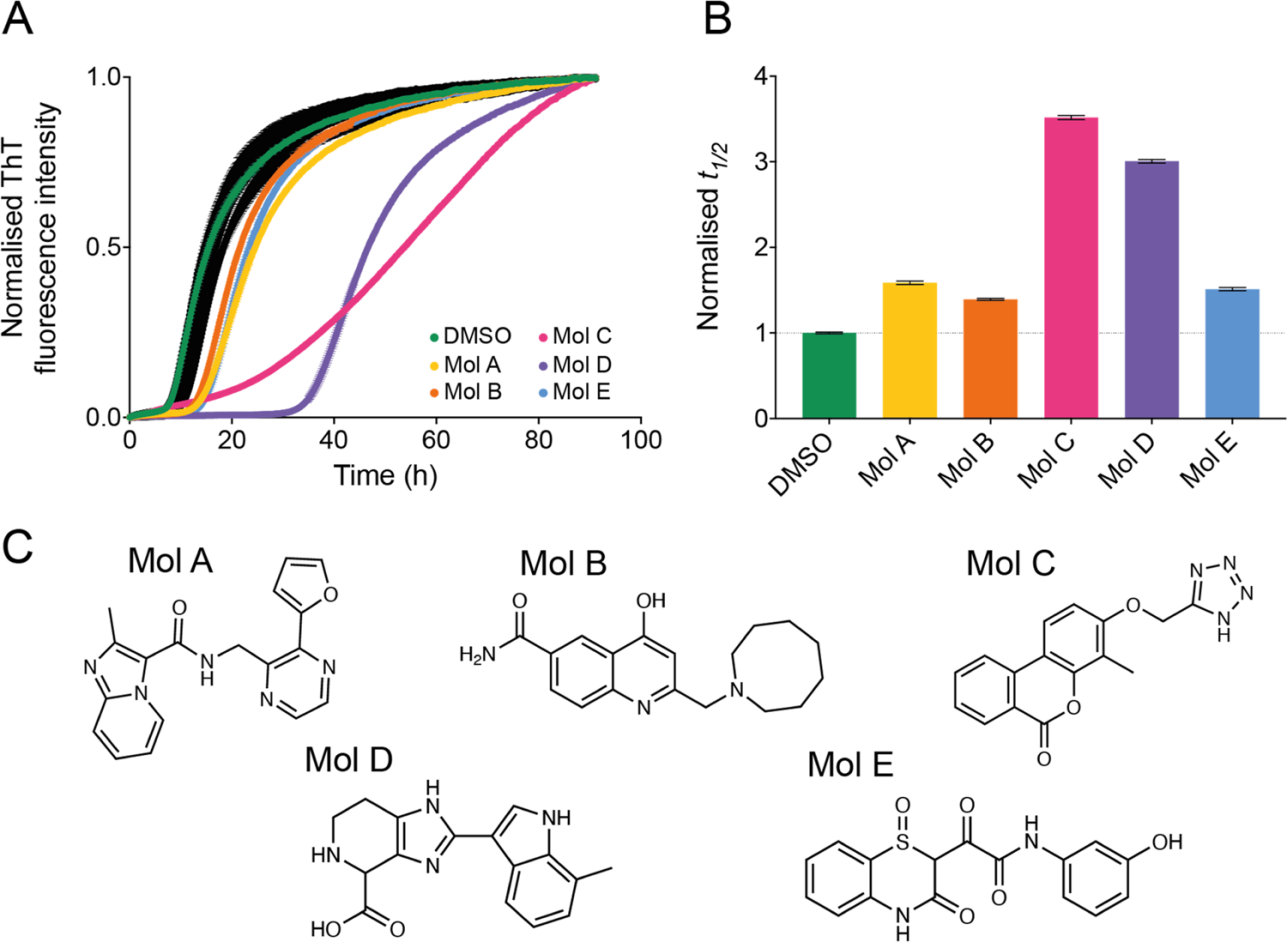
Five compounds selected from the docking library inhibit the aggregation of α-synuclein. (A) Kinetic profiles of a 10 μM solution of α-synuclein in the presence of 25 nM seeds at pH 4.8 and 37 °C, in the presence of 1% DMSO alone (beige), in the presence of 10 molar equivalents of compounds A–E (represented in different colors), or in the presence of 10 molar equivalents of other compounds in the docking library that did not affect significantly α-synuclein aggregation (black). (B) Relative *t*_1/2_ of the aggregation of α-synuclein in the presence of compounds A–E as shown in (A), normalized to the DMSO control. (C) Chemical structures of compounds A–E. Throughout, error bars represent mean ± SEM of two replicates.

**Figure 3:**
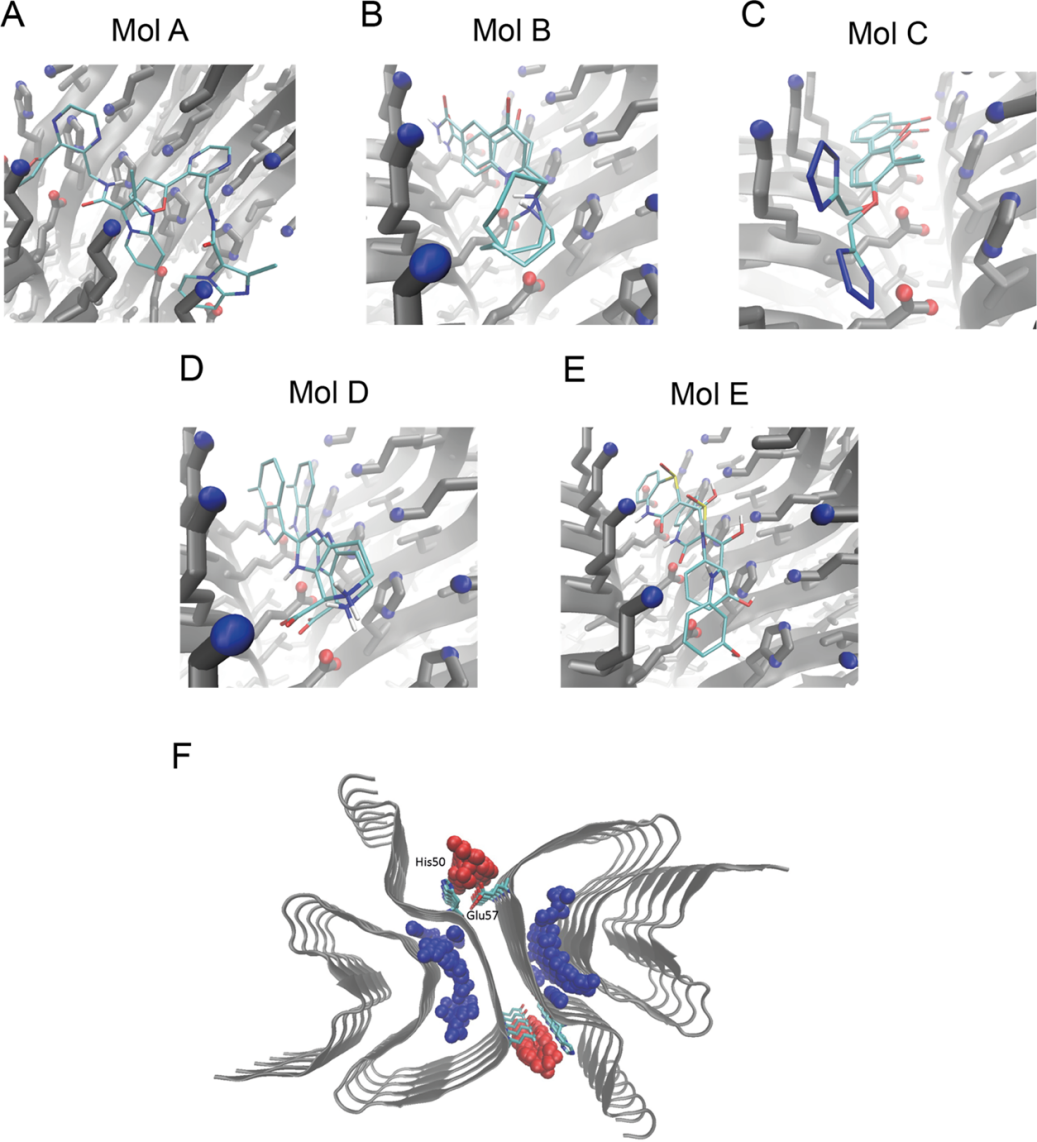
Computational docking of compounds to α-synuclein fibrils. (A–E) Binding poses of compounds A–E to the selected binding pocket in α-synuclein fibrils (centered between residues His50 and Glu57), determined either through FRED or AutoDock Vina. (F) Representation of possible binding pockets in the fibril structure (PDB: 6cu7, cyan) identified by Fpocket, with pockets in the fibril core (blue spheres), and at the fibril surface (red spheres). Key binding site residues His50 and Glu57 are shown in licorice representation.

From the library of centroids, we selected 67 compounds for experimental validation in terms of their binding affinity toward α-synuclein fibrils (Materials and Methods section). In particular, compounds were screened for their potency against 10 μM α-synuclein, which is relatively close to the estimated physiological concentration of α-synuclein in the neuronal synapse (≈50 μM).^45^ Out of the 67 compounds tested, five compounds were found to inhibit α-synuclein aggregation (Figure 2). The compounds were found to inhibit the aggregation of α-synuclein to different extents. In particular, three of the compounds (A, B, and E) showed moderate potency, increasing the half-time (*t*_1/2_) of the aggregation by 1.5 times, while compounds C and D exhibited stronger potency by increasing the *t*_1/2_ of the aggregation by 3.5 and 3 times, respectively. We also found that the chemical structures of these inhibitors tend to involve aromatic moieties (Figure 2C). More specifically, the aromatic regions of these compounds appeared within close proximity to the residues along the groove of the α-synuclein fibrils, suggesting that interactions could be established between the compounds and α-synuclein through these regions (Figure 3). Furthermore, we also observed a high similarity in terms of the compound positions within the selected groove of α-synuclein fibrils between using docking methods, thus suggesting that the binding of these compounds to the α-synuclein fibrils may involve specific interactions, which are likely to be a combination of electrostatic and nonpolar nature (Figure 3). Despite this observation, we also note the variety of polymorphic α-synuclein fibril structures that have been reported, which can differ in their structure, or the assembly of the protofilaments.^13−17^ Such diversity in the structural features of the α-synuclein fibrils formed may have contributed to the lower hit rate of compounds obtained from the *in silico* docking. Thus, further optimization by docking compounds across multiple polymorphic structures reported may increase the hit rate of positive compounds that are able to inhibit the aggregation of α-synuclein. A recent study has shown that the structures of α-synuclein fibrils derived from PD patients are different from the one used in this study.^46^ We anticipate that the approach that we describe in this work will be applicable to these new fibril structures, once an *in vitro* assay capable of reproducing them will be developed.

To rule out potential effects whereby the compounds inhibit the aggregation of α-synuclein by stabilizing nonfibrillar aggregates, we used transmission electron microscopy (TEM) to image the α-synuclein species formed at the end of the aggregation reaction in the absence and presence of compound C (Figure S2). These measurements showed the presence of α-synuclein fibrils at the end of the aggregation process both in the absence and presence of compound C, suggesting that the compounds that we identified are able to delay the aggregation process without redirecting it toward the formation of nonfibrillar α-synuclein aggregates (Figure S2).

### Kinetic Analysis of α-Synuclein Aggregation in the Presence of the Inhibitors

**Figure 4:**
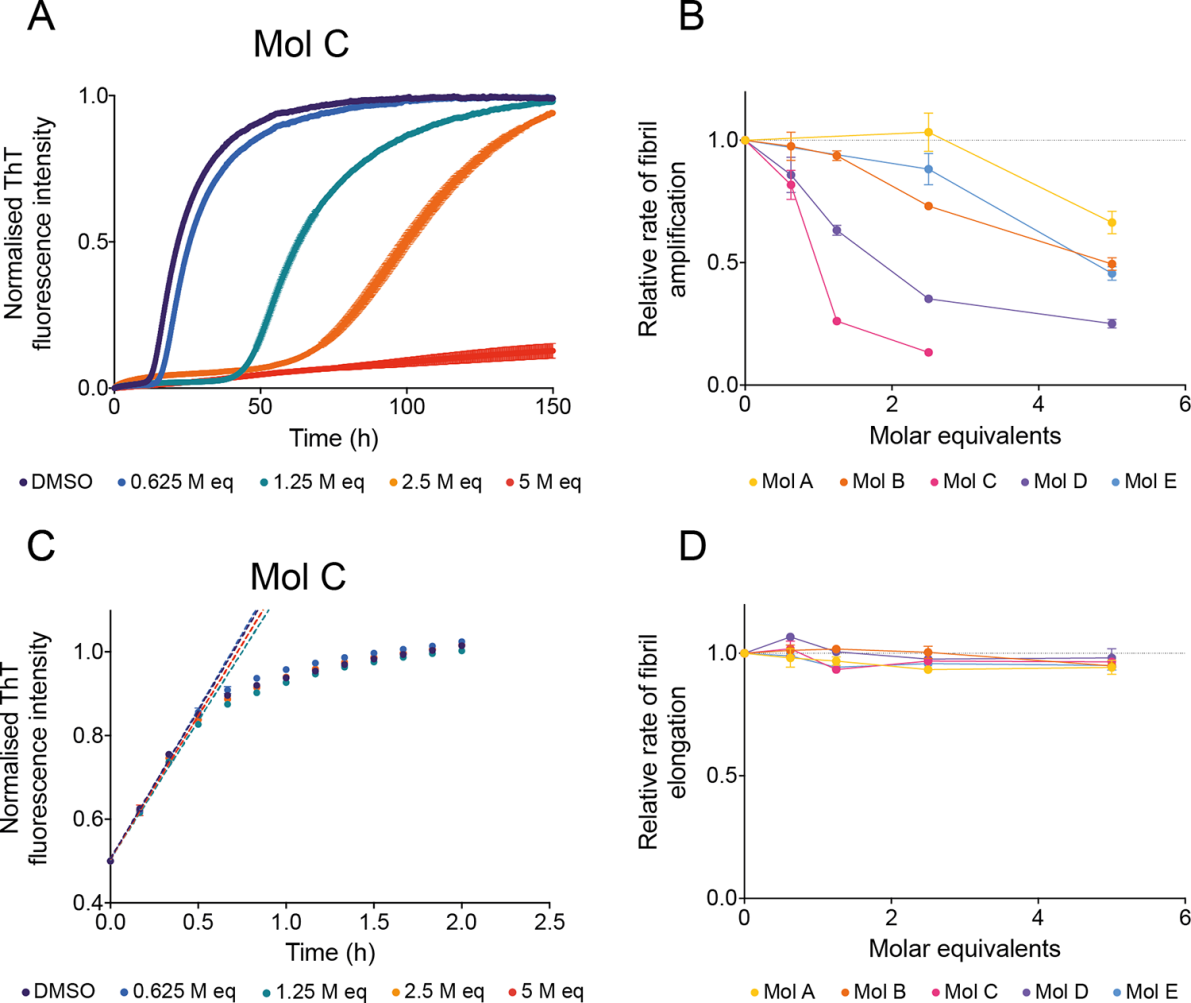
Compounds identified by docking specifically inhibit the proliferation of α-synuclein aggregates by secondary nucleation. (A) Kinetic profiles of a 10 μM solution of α-synuclein in the presence of 25 nM seeds at pH 4.8, 37 °C, in the presence of either 1% DMSO alone (purple) or increasing molar equivalents of compound C (represented in different colors). (B) Relative rate of fibril amplification of α-synuclein in the presence of compounds A–E as shown in (A) and Figure S3, normalized to the DMSO control. (C) Kinetic profiles of a 10 μM solution of α-synuclein in the presence of 5 μM seeds at pH 4.8, 37 °C, in the presence of either 1% DMSO alone (purple) or increasing molar equivalents of compound C (represented in different colors). Dotted lines indicate the *v*_max_ of the reaction which is used to extract the elongation rate of the aggregation process. (D) Relative rate of fibril elongation of α-synuclein in the presence of compounds A–E as shown in (C) and Figure S5,6, normalized to the DMSO control. Throughout, error bars represent mean ± SEM of three replicates.

To characterize the inhibitory potency of the five compounds against the aggregation of α-synuclein, we measured the secondary nucleation process of α-synuclein in the presence of varying compound concentrations, from substoichiometric ratios (0.625 molar equivalents) to overstoichiometric ratios (5 molar equivalents) (Figures 4A and S3–S5). For all of the five compounds, we observed a dose-dependent inhibition in the aggregation of α-synuclein, resulting in a systematic increase in the *t*_1/2_ of aggregation. Similarly to what we observed in the preliminary screening, the potency of the compounds varied between compounds A, B, and E, which exhibited a weaker effect, and compounds C and D, which had a stronger effect (Figures 4A and S3–S5). We further quantified the effect of the compounds, finding that they were able to significantly inhibit the rate of fibril amplification. This mechanism of action redirects the overall reactive flux toward elongation events, thus promoting the formation of fibrils of longer dimensions instead.^47^ We obtained these results by fitting the experimental data with a logistic function describing the amplification of α-synuclein aggregates over time (Materials and Methods section) (Figure 4B). These compounds are likely to compete with α-synuclein monomers on the nucleation sites, as shown previously with other compounds and molecular chaperones that also exhibit inhibition of secondary nucleation processes.^27,29,47,48^

The potency in inhibiting the amplification rate of the compounds was found to be in the order C > D > B and E > A. For instance, at 2.5 molar equivalents, while compounds C and D were able to inhibit the amplification rate of α-synuclein by 87 and 65%, respectively, compounds B and E were only able to inhibit this rate by 27 and 12%, respectively, and compound A was not able to significantly inhibit this process at this molar equivalent concentration.

To further probe the mechanism by which the compounds inhibit the aggregation process of α-synuclein, we also measured the aggregation process of α-synuclein in the absence and presence of the compounds with the addition of high concentration of preformed α-synuclein fibril seeds (Figures 4C, S5, and S6). Under such conditions, the aggregation process of α-synuclein proceeds as an exponential rather than sigmoidal function, indicative of an aggregation mechanism dominated by elongation processes with negligible contribution of secondary nucleation^35,41^ (Figure 4C). This phenomenon can be ascribed to a large number of growth-competent ends of fibrils present at the start of the aggregation process for further monomer addition. We observed that, under such conditions, the elongation rate, and consequently the aggregation process, was not significantly perturbed by the presence of the compounds (Figures 4C,D, S5, and S6). Thus, the inhibition, as observed using the low-seed aggregation assay, is likely due to the inhibition of the secondary nucleation process rather than the elongation process (Figures 4A, S3). Experimentally, the lack of significant effects on both the rate and amplitude of fluorescence emitted in the ThT-based assay under the high-seed conditions suggests that the compounds do not interfere with the fluorescence of ThT itself, and the inhibition as observed from the low-seed aggregation assay is likely to be a specific perturbation in the aggregation of α-synuclein (Figure S5). This also indicates that the compounds that were identified using our approach are likely binding the surface of α-synuclein fibrils, rather than the ends, further supporting their binding to the selected groove. Finally, compounds previously found to interact directly with α-synuclein monomers have been shown to interfere with all microscopic steps in the aggregation process, i.e., secondary nucleation and elongation, as the concentration of free α-synuclein is reduced.^49^ Since the five compounds identified here do not affect greatly the elongation process, their interaction with α-synuclein monomers is unlikely to be significant. Further insights into their mechanism of action could be obtained from more extensive structural studies, particularly at different pH conditions, which also alters the significance of secondary nucleation in the overall aggregation process of α-synuclein.^35^

### Compound C Inhibits α-Synuclein Oligomer Formation

**Figure 5:**
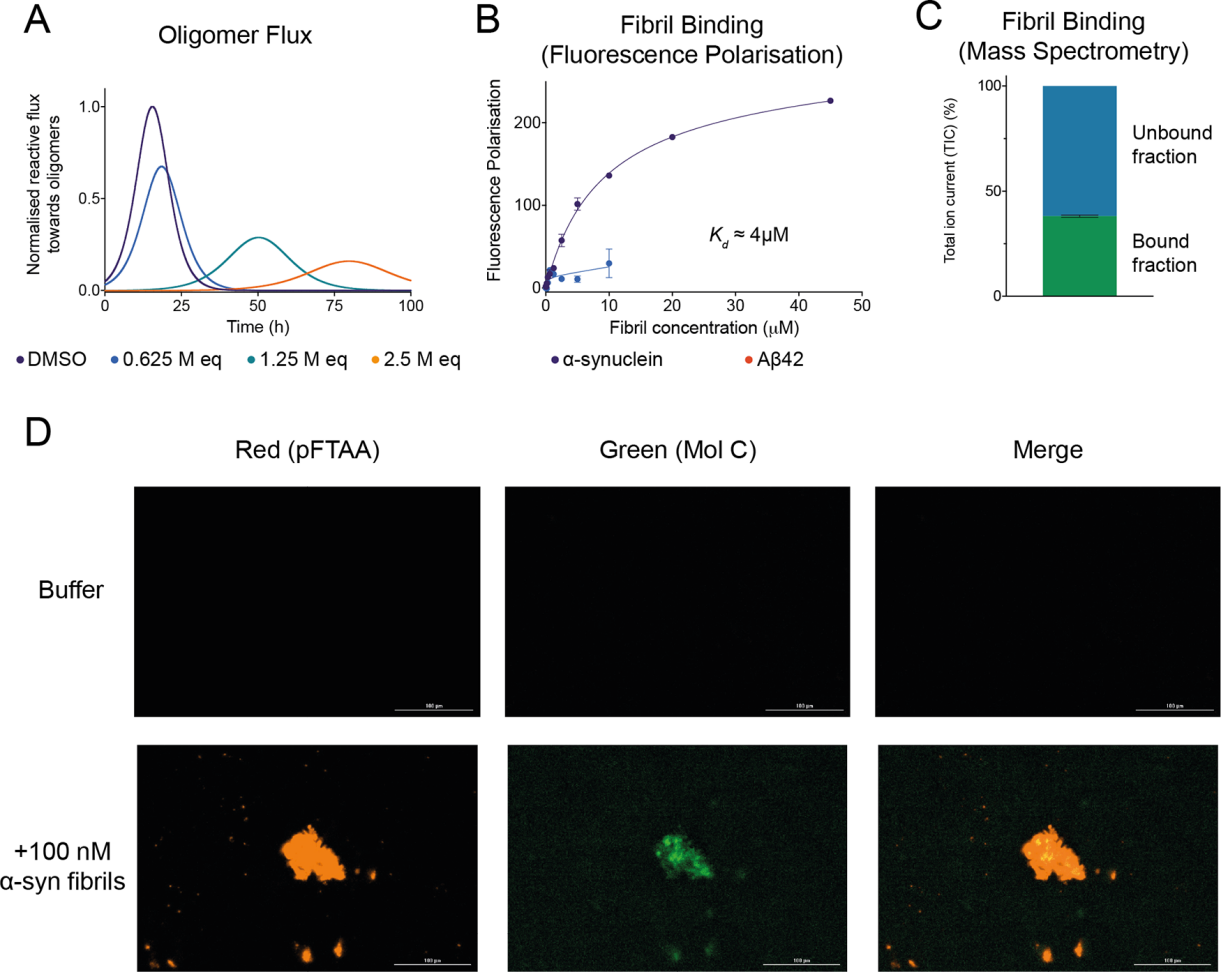
Compound C inhibits the reactive flux toward α-synuclein oligomers and displays binding affinity and specificity toward α-synuclein fibrils. (A) Time dependence of the reactive flux toward α-synuclein oligomers either in the presence of 1% DMSO alone (purple) or in the presence of increasing molar equivalents of compound C (represented in different colors), normalized to the DMSO control. (B) Change in fluorescence polarization (in mP units) of 10 μM compound C with increasing concentrations of either α-synuclein fibrils (purple) or Aβ42 fibrils (red). The solid lines are fits to the points using a one-step binding curve, estimating a *K*_d_ of 4 μM for compound C toward α-synuclein fibrils. (C) Total ion current (TIC) of 10 μM compound C bound and unbound to 10 μM α-synuclein fibrils detected by mass spectrometry (see the Materials and Methods section). (D) Representative images indicating either the fluorescence of the red channel (amyloid-specific dye pFTAA) or the green channel (compound C) following incubation in the absence (top) or presence (bottom) of 100 nM α-synuclein fibrils. Throughout, error bars represent mean ± SEM of two replicates.

The reactive flux toward α-synuclein oligomers in the aggregation reaction of α-synuclein is governed by multiple processes, including crucially secondary nucleation.^31^ By extracting the change in the amplification rate due to the presence of the compounds, and by accounting for the specific inhibition of the secondary nucleation process rather than the elongation process, we can calculate the reactive flux toward oligomers over time in the absence and presence of increasing concentrations of the compounds (Figures 5A and S7) (Materials and Methods section). Depending on their potency, we observed that compounds were able to delay the rate of reactive flux toward oligomers, as well as a delay in the overall formation of oligomers over time (integral area of flux).^28,33^ Future studies will be required to experimentally validate this drop in the reactive flux toward oligomers, as demonstrated for anti-Aβ antibodies previously using a combination of size-exclusion chromatography and mass spectrometry.^50^

### Compound C Binds α-Synuclein Fibrils

As a validation of the binding of these candidates to the surface of α-synuclein fibrils, we also performed fluorescence polarization experiments of compound C, the strongest inhibitor of all of the positive compounds, in the presence of increasing concentrations of α-synuclein fibrils (Figures 5B and S7). Additionally, since compound C exhibits a high degree of intrinsic fluorescence, fluorescence polarization measurements allowed us to determine the proportion of bound and unbound fractions of compound C toward α-synuclein fibrils (Figure S8). We observed a dose-dependent increase in the polarization (a unit-less property) of compound C as a function of increasing concentrations of α-synuclein fibrils. By fitting this response as a function of the concentration of α-synuclein fibrils, the apparent dissociation constant (*K*_d_) was found to be about 4 μM (Figure 5B) (Materials and Methods section). However, we note that the *K*_d_ value obtained is based on the concentration of α-synuclein fibrils in monomer equivalents. Since α-synuclein fibrils tend to contain at least 200 monomers per unit,^35^ it is thus likely that the actual *K*_d_ value (in terms of number of fibrillar units) should be significantly lower, considering the binding of compound C targets a distinct groove within α-synuclein fibrils rather than amino acid sequences within individual monomeric subunits (Figure 3C).

To test for the specificity of compound C for α-synuclein fibrils, we measured the change in polarization upon incubation of compound C with Aβ42 fibrils, which are associated with Alzheimer’s disease (Figure 5B). In this case, we observed a much lower increase in the polarization, as only a slight increase could be measured at 10 μM Aβ42 fibrils. This result suggests that compound C binds specifically to the surface of α-synuclein fibrils, as predicted from the docking, rather than having generic nonspecific interactions with hydrophobic aggregates (Figure 5B).

To further support the fluorescence polarization data, we also performed a mass-spectrometry-based pull-down assay to assess the amounts of compound bound to α-synuclein fibrils (Figure 5C) (Materials and Methods section). We found that ∼40% of compound C was still associated with α-synuclein fibrils after an ultracentrifugation pull-down (Figure 5C). Finally, we sought to explore the potential use of compound C as a fluorescent probe of α-synuclein fibrils. In this experiment, fluorescent images of α-synuclein fibrils were acquired after incubation with compound C (through the green channel) or the amyloid-specific fluorescence dye pFTAA (through the red channel) (Figure 5D). We observed a distinct overlap of the fluorescence between both channels, demonstrating the colocalization of compound C with the α-synuclein fibrils (stained by pFTAA). This confirms the significant affinity of compound C toward α-synuclein fibrils as previously shown through chemical kinetics. We further visualized the colocalization of compound C with exogenous α-synuclein fibrils in the presence of neuroblastoma cells (Figure S9). Indeed, the same behavior manifested as well, suggesting the higher specific affinity of compound C toward the α-synuclein aggregates over other cellular material. This also shows the potential use of such compounds in biological studies involving α-synuclein aggregation.

## Discussion

In this work, we have reported the use of a structure-based approach to identify small molecules whose mechanism of action is to bind α-synuclein fibrils and inhibit the secondary nucleation step in the autocatalytic proliferation of α-synuclein aggregates. We anticipate that such an approach will enable the rational design and systematic development of small molecules capable of binding specific sites with different properties on α-synuclein fibrils of different morphologies, as well as in fibrils formed by other disease-related proteins, thereby creating new opportunities in the diagnostics and therapeutics for protein misfolding diseases.

## Supporting Information Available

Predicted binding affinity of compounds via computational docking; TEM images of α-synuclein fibrils formed in the presence of molecules; kinetic profiles of α-synuclein aggregation in the presence of molecules; time dependence of reactive flux toward α-synuclein oligomers in the presence of molecules; fluorescence emission spectra of molecule C; colocalization of molecules with α-synuclein fibrils in the presence of neuroblastoma cells; list of all molecules from docking studies experimentally validated; and supplementary methods, supplementary figures, and supplementary table (PDF)

## Abbreviations

*K*_d_: apparent dissociation constant
SAR: structure–activity relationship
TEM: transmission electron microscopy
