# Supplementary material for: Structure-Based Discovery of Small-Molecule Inhibitors of the Autocatalytic Proliferation of α-Synuclein Aggregates
Source: Mol Pharm. 2022 Nov 14;20(1):183–93. doi: 10.1021/acs.molpharmaceut.2c00548 (PMC9811465; doi:10.1021/acs.molpharmaceut.2c00548)
Supplement: Supplementary file 1 — mp2c00548_si_001.pdf [file mp2c00548_si_001.pdf]

Supplementary Information

Structure-based discovery of small molecule inhibitors of the autocatalytic proliferation of α-synuclein aggregates

Sean Chia[1+], Z. Faidon Brotzakis[1], Robert I. Horne[1], Andrea Possenti[1], Benedetta Mannini[1], Rodrigo Cataldi[1], Magdalena Nowinska[1], Roxine Staats[1], Sara Linse[2], Tuomas P. J. Knowles[1,3], Johnny Habchi[1], Michele Vendruscolo[1]*

[1]*Centre for Misfolding Diseases, Yusuf Hamied Department of Chemistry, University of Cambridge, Cambridge CB2 1EW, UK*

[2]*Department of Biochemistry & Structural Biology, Center for Molecular Protein Science, Lund University, 221 00 Lund, Sweden*

[3]*Department of Physics, Cavendish Laboratory, Cambridge CB3 0HE, UK*

* To whom correspondence should be addressed. Email: mv245@cam.ac.uk

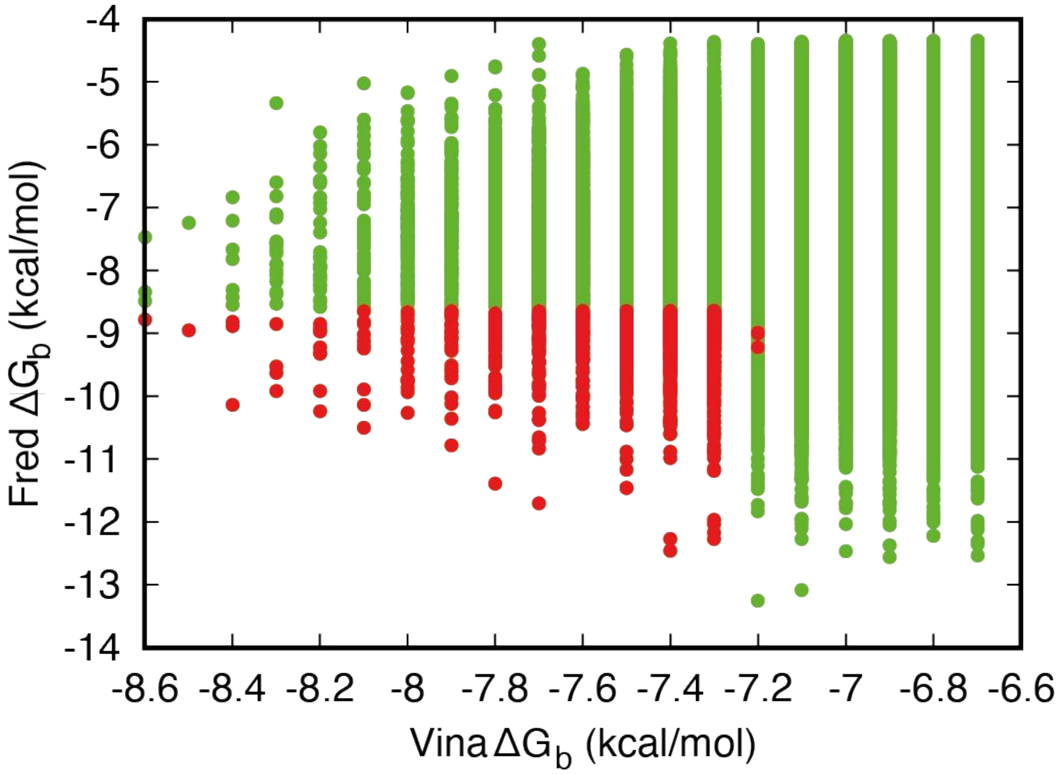

Figure S1. Distribution of the predicted binding affinity for α-synuclein fibrils of the top 10,000 compounds screened by computational docking. The predicted binding score (ΔG_b) of each compound calculated either via FRED (Fred) or AutoDock Vina (Vina). Points in red denote the top 1,000 compounds with the highest predicted binding scores calculated by both methods, which were further selected as an enriched compound library of potential α-synuclein fibril binders.

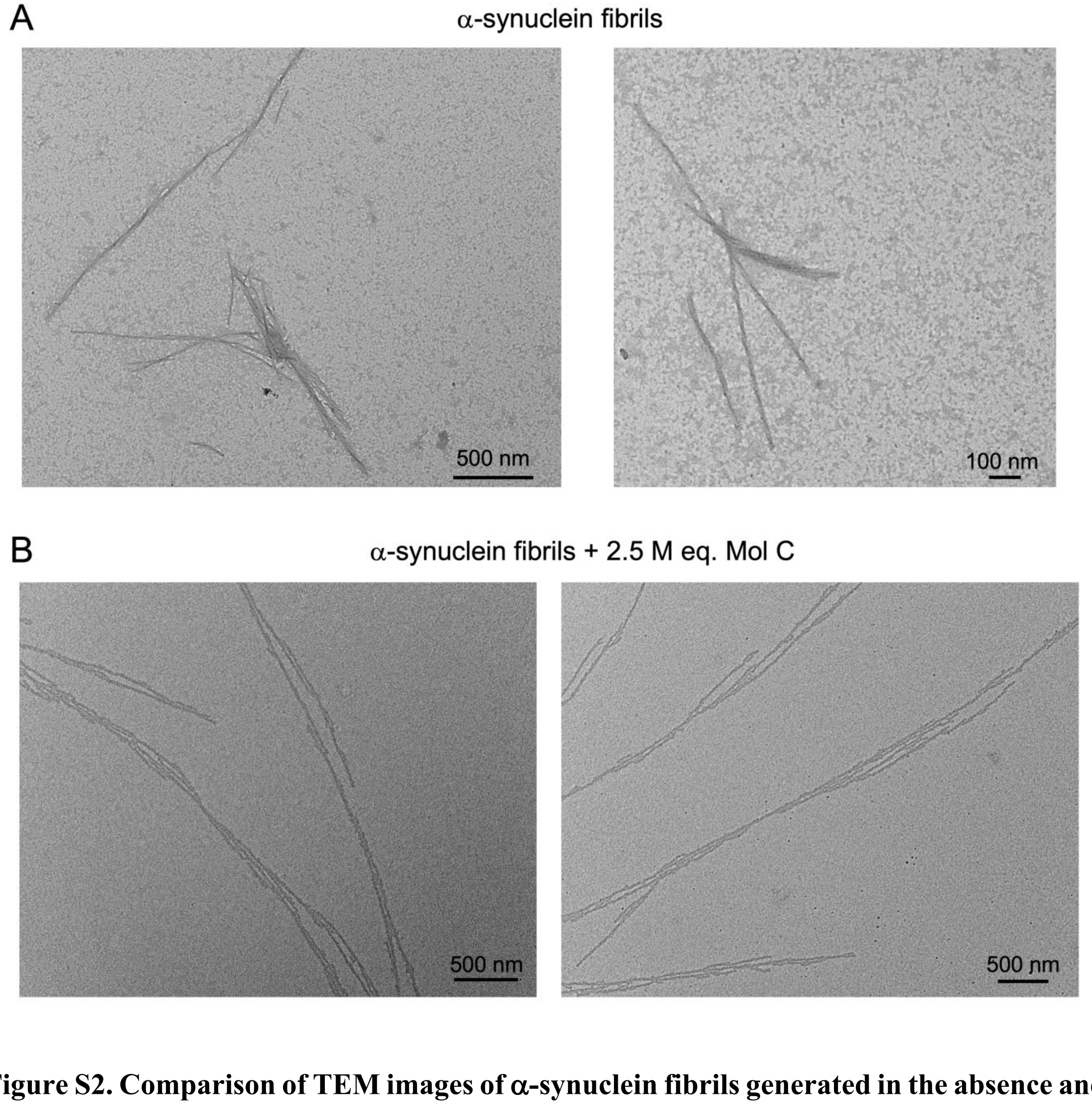

Figure S2. Comparison of TEM images of α-synuclein fibrils generated in the absence and presence of compound C, which inhibits α-synuclein aggregation. (A,B) TEM images of 10

μM α-synuclein fibrils grown in the absence (**A**) or presence (**B**) of 2.5 molar equivalents (M. eq) of compound C.

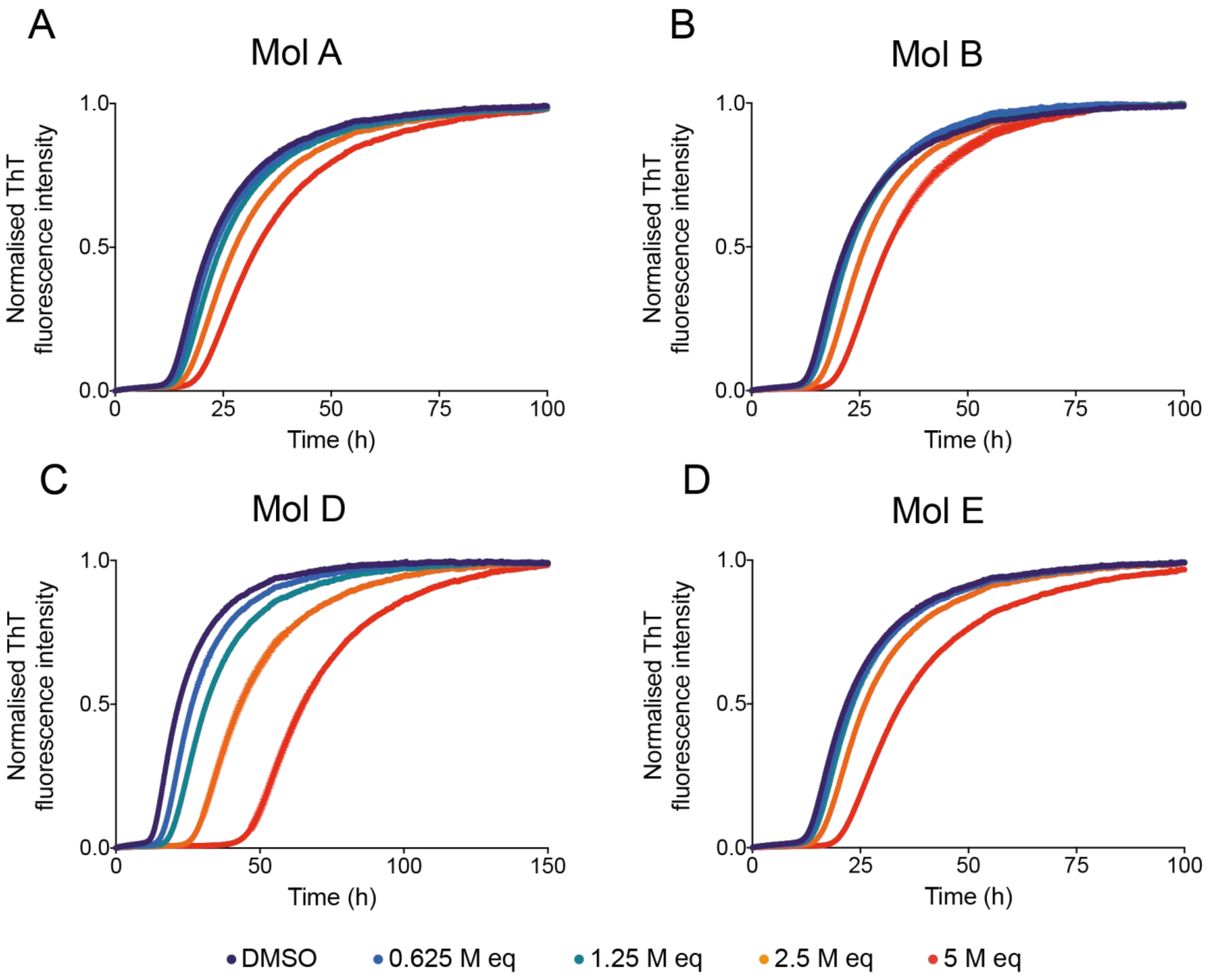

Figure S3. Positive compounds from the docking library inhibit the surface-catalysed secondary nucleation process of α-synuclein aggregation. (A-D) Kinetic profiles of a 10 μM solution of α-synuclein in the presence of 25 nM seeds at pH 4.8, 37 ^{o}C, either in the presence of 1% DMSO alone (purple), or in the presence of increasing molar equivalents of either compound A (**A**), compound B (**B**), compound D (**C**), or compound E (**D**). Throughout, error bars represent means ± SEM of three replicates.

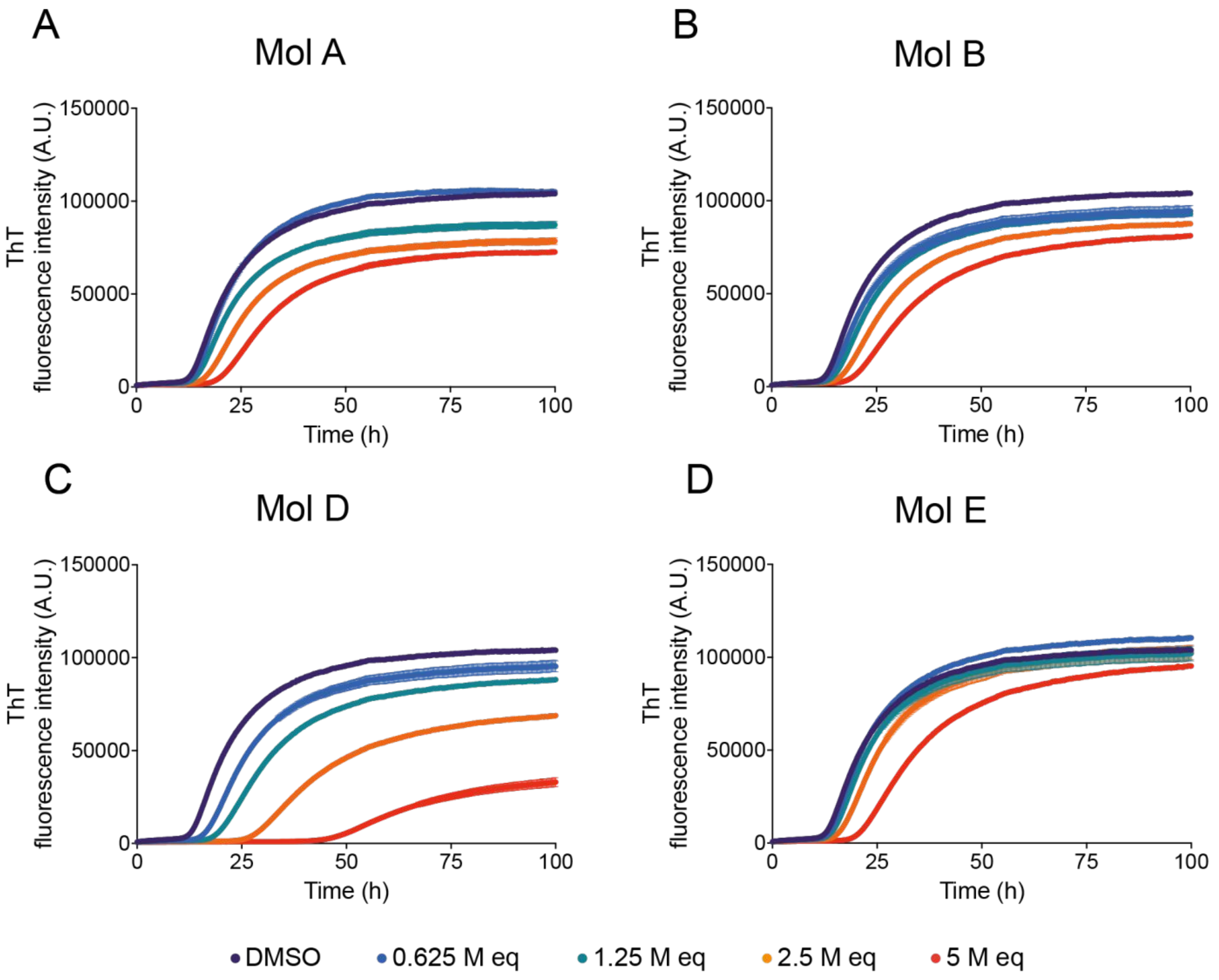

Figure S4. Raw ThT fluorescence traces of α-synuclein aggregation in the presence of positive compounds from the docking library. (A-D) Raw ThT fluorescence traces over time of a 10 μM solution of α-synuclein in the presence of 25 nM seeds at pH 4.8, 37 ^{o}C, either in the presence of 1% DMSO alone (purple), or in the presence of increasing molar equivalents of either compound A (**A**), compound B (**B**), compound D (**C**), or compound E (**D**). Throughout, error bars represent means ± SEM of three replicates.

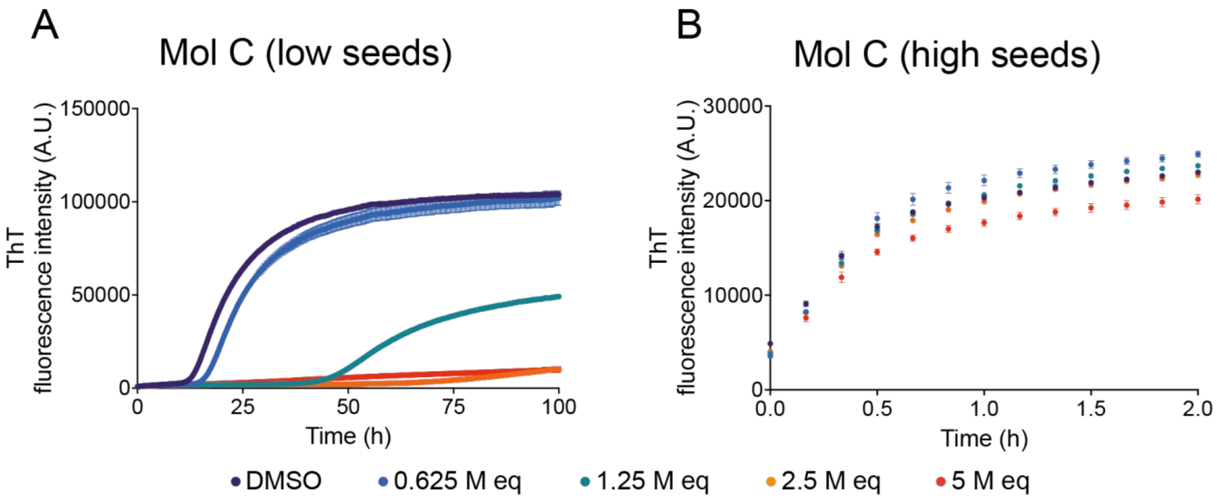

Figure S5. Raw ThT fluorescence traces of α-synuclein aggregation in the presence of compound C. (A-B) Raw ThT fluorescence traces over time of a 10 µM solution of α-synuclein in the presence of either 25 nM (**A**) or 5 µM (**B**) α-synuclein seeds at pH 4.8, 37 °C, either in the presence of 1% DMSO alone (purple), or in the presence of increasing molar equivalents (M. eq) of compound C (represented in different colours). Throughout, error bars represent means ± SEM of three replicates.

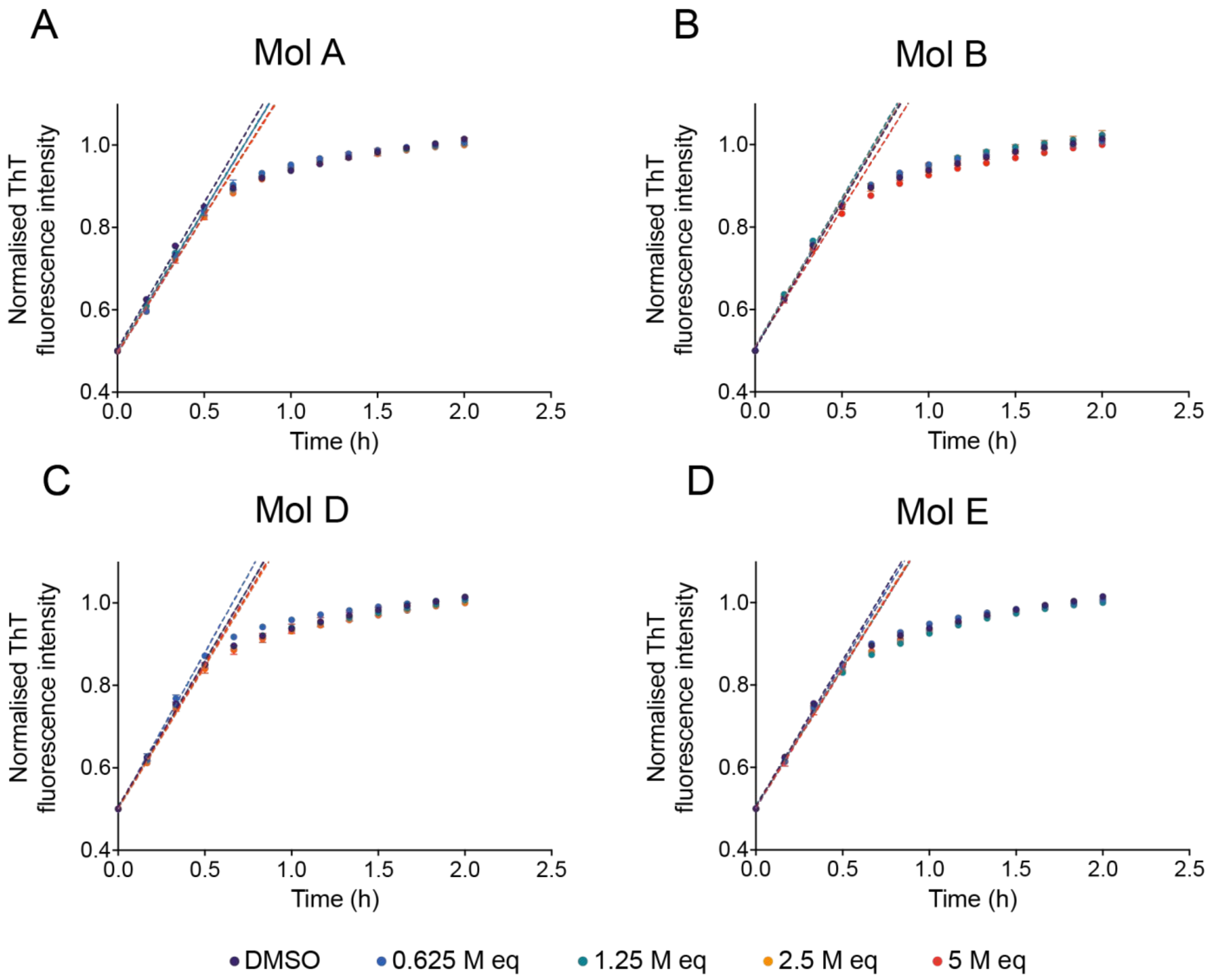

Figure S6. Positive compounds from the docking library do not significantly inhibit the elongation process of α-synuclein aggregation. (A-D) Kinetic profiles of a 10 μM solution of α-synuclein in the presence of 5 μM seeds at pH 4.8, 37°C, either in the presence of 1% DMSO alone (purple), or in the presence of increasing molar equivalents of either compound A (**A**), compound B (**B**), compound D (**C**), or compound E (**D**). Dotted lines indicate the v_{max} of the reaction which is used to extract the elongation rate of the aggregation process (**Fig. 4D**). Throughout, error bars represent means ± SEM of three replicates.

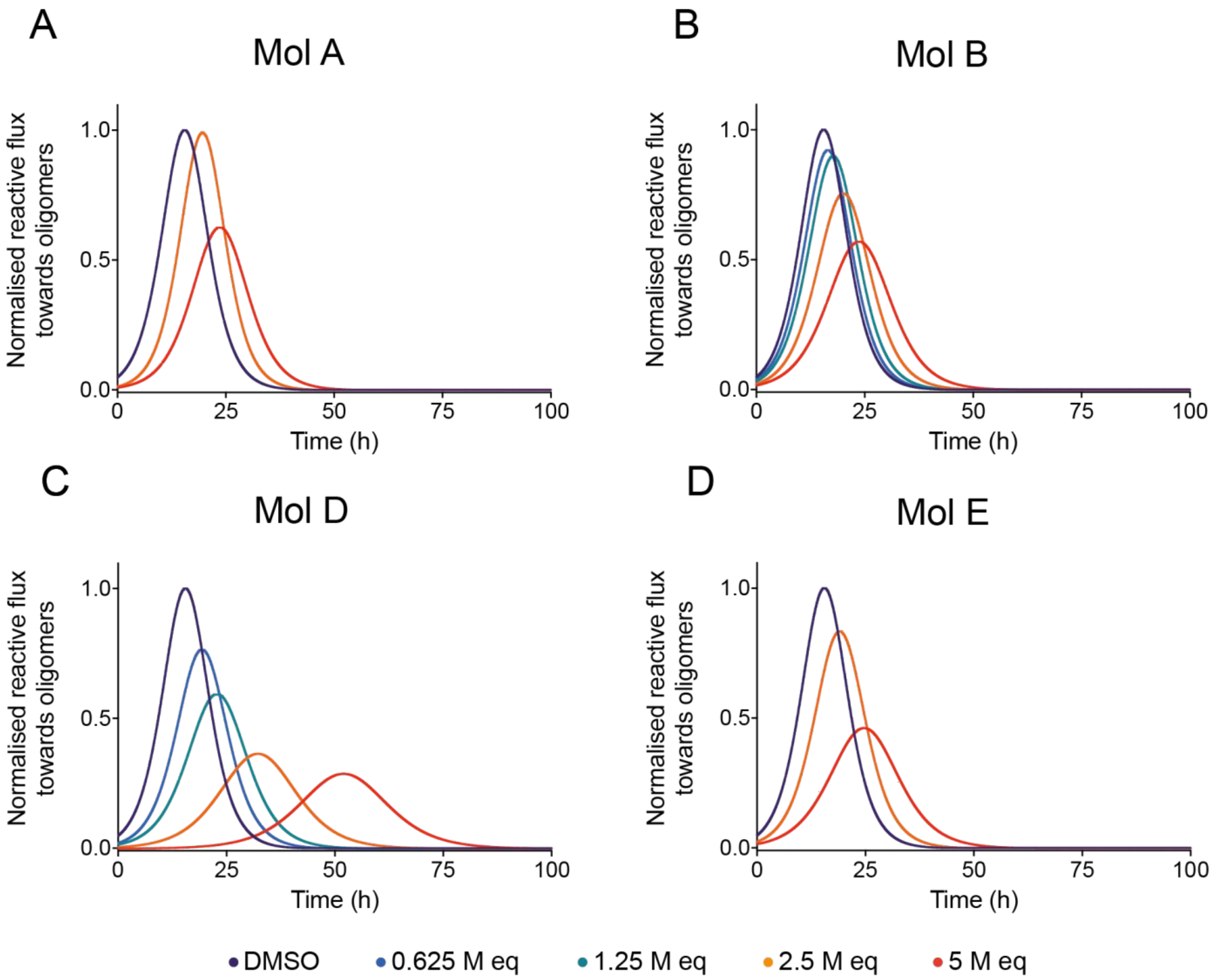

Figure S7. Positive compounds from the docking library reduce the reactive flux towards α-synuclein oligomers. (A-D) Time dependence of the reactive flux towards α-synuclein oligomers either in the presence of 1% DMSO alone (purple) or in the presence of increasing molar equivalents (M eq) of compound A **(A)**, compound B (**B**), compound D (**C**), or compound E (**D**), normalised to the DMSO control.

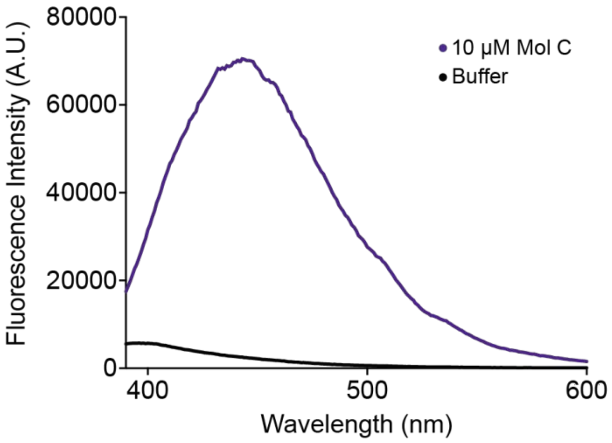

Figure S8. Fluorescence emission spectra of compound C. Fluorescence emission spectra of either buffer (black) or 10 μM compound C (purple) in sodium phosphate buffer, pH 4.8, 1 mM EDTA (λ_{ex} = 360 nm).

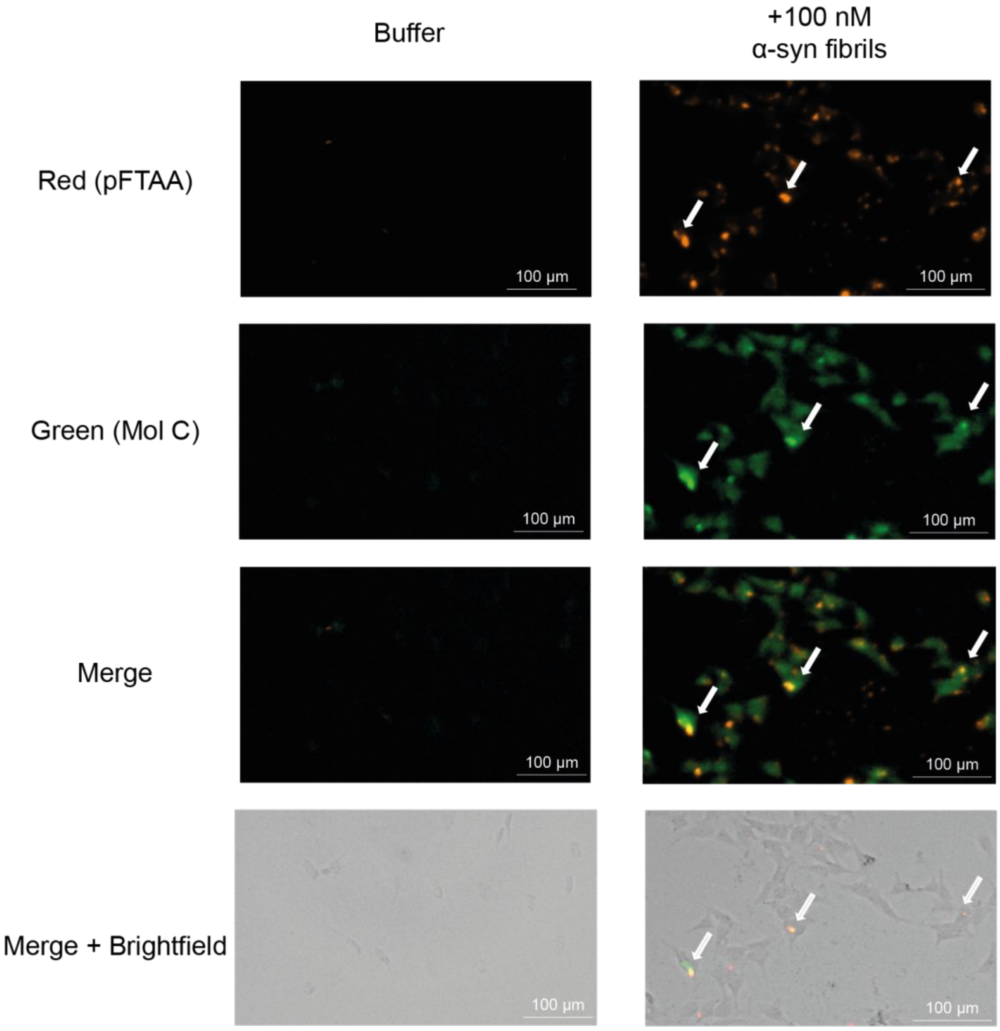

Figure S9. Co-localisation of compound C with α-synuclein fibrils in the presence of neuroblastoma cells. Representative images indicating either the fluorescence of the red channel (amyloid-specific dye pFTAA) or the green channel (compound C) following incubation in the absence (top) or presence (bottom) of 100 nM α-synuclein fibrils with neuroblastoma cells. White arrows indicate the specific co-localisation of compound C with α-synuclein fibrils in the presence of cells.

	**SMILES of experimentally validated compounds**
1	Cc(n1)c(C(NCc2c(c3occc3)nccn2)=O)n4c1cccc4 – *Compound A*
2	NC(c(cc1)cc2c1nc(CN3CCCCCCC3)cc2O)=O – *Compound B*
3	Cc(c(OCc1[nH]nnn1)cc2)c3c2c(cccc4)c4c(=O)o3 – *Compound C*
4	Cc(ccc1)c2c1c(c3[nH]c4c(C(C(O)=O)NCC4)n3)c[nH]2 – *Compound D*
5	Oc1cc(NC(C(C2C(Nc3c(S2=O)cccc3)=O)=O)=O)ccc1 – *Compound E*
6	O=C(N1CCC2(CC1)CCO2)Cc(n[nH]3)c4c3cccc4
7	O=C(N1CCN(c(ncc[nH]2)c2=O)CC1)C3CCc4c(O3)cccc4
8	CCN1C(NC2(C1=O)CCN(C(c3c(F)cccc3)=O)CC2)=O
9	OC(C1COCCN1C(Cn(ncc2=O)c3c2cccc3)=O)=O
10	COc1cc(CC(NC2CCN(c3ccccc3)C2=O)=O)ccc1
11	OC(C1CC2CCCCC2N1C(c(cn3)nc4c3cccc4)=O)=O
12	CC(O)(c1cc(c2cc(C(N3CCOCC3)=O)ccc2)cnc1)C
13	NC(CN1CCc2c(C1)c(c3ccc(c4ccccc4)cc3)n[nH]2)=O
14	CC1CN(c2ccc(C)cc2)C(CN1C(c(cnc[nH]3)c3=O)=O)=O
15	Cc1c(F)cc(NC(N2CCN(CC2)Cc3cnccc3)=O)cc1
16	Cc1[nH]c2c(CC(C(N3CCOC4(C3)CCCCC4)=O)CC2)n1
17	CCc1n(CC(N2CC[C@@H]3OCc4n([C@H]3C2)nnc4)=O)ccn1
18	O=C(c1n[nH]cn1)N2CCN(C(c3c4c(CCCC4)cs3)=O)CC2
19	Cc(cc(=O)n1CCN2CCOC(c3ccccc3)C2)[nH]c1=O
20	CN(C(c(cc1)cc2n1cnn2)=O)C3CCCc4c3cccc4
21	O=C(C(n1nnnc1)c2ccccc2)NCCn(cn3)c4c3cccc4
22	CN(C(c(cnc(C)[nH]1)c1=O)=O)Cc(cc2)nc3c2cccc3
23	Cc(no1)c2c1ncnc2N3CCCC(c(nn4)n5c4cccc5)C3
24	CN1[C@@H](c2ccncc2)[C@@H](CC1=O)CNC3Cc4c(C3)cccc4
25	O=c([nH]n(c(nnc1)c2c1cccc2)c3=O)c4c3cccc4
26	O=C1CCCC2=C1C(n3c(N2)nnn3)c4ccccc4
27	CC1CC(c2c(N1C(Cc(no3)c4c3cccc4)=O)cccc2)C(N)=O
28	COc1c2c(c3c(CC2)c(C(n4cncc4)=O)n[nH]3)ccc1
29	O=C(NCc1ncccc1)CN2C(c(ccc3)c4c3cccc24)=O
30	Cc1c(S(C)(=O)=O)cc(C(Nc(nn2)n3c2cccc3)=O)cc1
31	OC1(c2ccccc2)CCN(c3c4c(CCNCC4)ncn3)C1
32	CC1CC(C(O1)=O)N2C(NC3(C2=O)CCc4c3cccc4)=O
33	O=C(c1[nH]ccc1)Cn(cnc2c3cnn2c4ccccc4)c3=O
34	Oc(n1)c(CNC2CCCCNC2=O)cc3c1cccc3
35	CC1COCCN1C(c2c(C)c3c(CC(C)(CC3=O)C)[nH]2)=O
36	O=C(c(cc(cccc1)c1o2)c2=O)Nc3c4c(CCC4)no3
37	CC(N1CCN(C(c2[nH]cc(C)c2)=O)[C@H]3CS(=O)(C[C@@H]13)=O)=O
38	O=C(C1CC12CCCc3c2cccc3)NS(=O)(c4cnccc4)=O
39	CC1CC(c(cn2)c(C1)c3c2nc(N4CCCCC4)[nH]c3=O)=O
40	CN1CCC2(CN(C(Cc(c(C)[nH]c(=O)[nH]3)c3=O)=O)CCN2C)CC1
41	Cc1n(c2cc(NC(Cn(ccc(C)c3)c3=O)=O)c(F)cc2)nnn1

42	O=C1CCC2(CN1CCCn(nn3)c4c3cccc4)CCOC2
43	O=C(C1CN(C(C1)=O)C2CCCC2)NC34CC5CC(C4)CC(C3)C5
44	CN1C(c2c(C1=O)cc(NC(C3CCC=CC3)=O)cc2)=O
45	Cc1cc(n(ccc(c2nn3)n4c3ncn4)c2=O)c(C)cc1
46	Fc1cc(n2nnnc2)c(C(Nc3cc4c(OCCO4)cc3)=O)cc1
47	Cc(cc1O)nc2n1nc(C34CC5CC(C4)CC(C3)C5)n2
48	C1(c2n3c(CCCCC3)nn2)CCCN(c(ncn4)c5c4nc[nH]5)C1
49	NC1(C2CC3CC(CC1C3)C2)CNC(Nc(cc4)cc5n4ccn5)=O
50	COc(cc1)cc2c1[nH]c3c2CCNC34CCN(CC4)C
51	CC(c1ccc(C(NN2C(C3C4CCC(C3C2=O)O4)=O)=O)cc1)C
52	CCCC12CCCN1C(N(C2=O)C3CCN(c4nnccc4)CC3)=O
53	OC12CCCCC1CN(C(C3(n4nnnc4)CCCCC3)=O)CC2
54	NC(c1ccccc1)CNS(=O)(c(ccc2)c3c2nccc3)=O.Cl
55	Fc1c(/C=C2Sc3n(C\2=O)c(c4ccccc4)nn3)cccc1
56	Cn(c(ncc(C(c1c(O)ccc(Cl)c1)=O)c2)c2c(=O)n3C)c3=O
57	O=c1c(C#N)c(c2cnccc2)c(COc3c4cccc3)c4[nH]1
58	O=C1/C(NS(=O)(c2c1cccc2)=O)=C/c3cc4c(OCO4)cc3
59	O=C1CNC(C2CN(CCN12)Cc3cc(Cc4c5cccc4)c5cc3)=O
60	Cc1c(N2C(C(C2=O)N3C(c4c(C3=O)cccc4)=O)C=O)cccc1
61	O=c([nH]1)oc2c1ccc(S(=O)(N3CCC4(CCCNC4)C3)=O)c2
62	NCC1Cc(c(O1)cc2)c3c2cc(c(ccc[nH]4)c4=O)cc3
63	O=C1NN(c2ccccc2)C(/C1=C/c(coc3c4cccc3)c4=O)=O
64	CC1CC1C(NCCC(N(C2C3CC4CC(CC2C4)C3)C)=O)=O
65	Cc1c(c2nc(c(cc3)cc4c3[nH]c(=O)[nH]4)on2)c5c(CNCC5)cn1
66	Fc(cc1F)cc2c1nc(c3nnn(C4CCNCC4)c3)[nH]2
67	Cc1c(CCNC(P(O)(O)=O)c2cc(C#N)ccc2)cccc1

Table S1. List of compounds from docking studies that were experimentally validated. The compounds are provided in the simplified molecular-input line-entry system (SMILES) format.
